# In Situ Assessment of Intrinsic Strength of X-I⋯OA-Type Halogen Bonds in Molecular Crystals with Periodic Local Vibrational Mode Theory

**DOI:** 10.3390/molecules25071589

**Published:** 2020-03-30

**Authors:** Yunwen Tao, Yue Qiu, Wenli Zou, Sadisha Nanayakkara, Seth Yannacone, Elfi Kraka

**Affiliations:** 1 Department of Chemistry, Southern Methodist University, 3215 Daniel Avenue, Dallas, TX 75275-0314, USA; yunwent@smu.edu (Y.T.); snanayakkara@smu.edu (S.N.); syannacone@smu.edu (S.Y.); 2 Grimwade Centre for Cultural Materials Conservation, School of Historical and Philosophical Studies, Faculty of Arts, University of Melbourne, Parkville, VIC 3052, Australia; yueq1@student.unimelb.edu.au; 3 Institute of Modern Physics, Northwest University, and Shaanxi Key Laboratory for Theoretical Physics Frontiers, Xi’an 710127, China; wzou@smu.edu

**Keywords:** halogen bonding, dihalogen, local vibrational mode theory, local stretching force constant, molecular crystal, chemical bond strength, vibrational spectroscopy, crystal engineering, generalized Badger’s rule, VASP

## Abstract

Periodic local vibrational modes were calculated with the rev-vdW-DF2 density functional to quantify the intrinsic strength of the X-I⋯OA-type halogen bonding (X = I or Cl; OA: carbonyl, ether and *N*-oxide groups) in 32 model systems originating from 20 molecular crystals. We found that the halogen bonding between the donor dihalogen X-I and the wide collection of acceptor molecules OA features considerable variations of the local stretching force constants (0.1–0.8 mdyn/Å) for I⋯O halogen bonds, demonstrating its powerful tunability in bond strength. Strong correlations between bond length and local stretching force constant were observed in crystals for both the donor X-I bonds and I⋯O halogen bonds, extending for the first time the generalized Badger’s rule to crystals. It is demonstrated that the halogen atom X controlling the electrostatic attraction between the σ-hole on atom I and the acceptor atom O dominates the intrinsic strength of I⋯O halogen bonds. Different oxygen-containing acceptor molecules OA and even subtle changes induced by substituents can tweak the $n\to \sigma^{*}$(X-I) charge transfer character, which is the second important factor determining the I⋯O bond strength. In addition, the presence of the second halogen bond with atom X of the donor X-I bond in crystals can substantially weaken the target I⋯O halogen bond. In summary, this study performing the in situ measurement of halogen bonding strength in crystalline structures demonstrates the vast potential of the periodic local vibrational mode theory for characterizing and understanding non-covalent interactions in materials.

## 1. Introduction

Halogen bonding (D-X⋯Y) is one important type of non-covalent interaction between a donor halogen atom X (F, Cl, Br or I) and an electron-rich atom/group Y (e.g., atoms with lone pair electrons including N, O, P and S) [1]. With its great bond strength tunability, halogen bonding has gained popularity in drug design [2,3,4,5], enzyme engineering [6], material science [7,8,9], catalysis [10,11,12,13] and crystal engineering [14,15,16]. The currently well-accepted understanding of halogen bonding focuses on the interplay of the following contributions: (1) charge transfer from nucleophile Y to the $\sigma^{*}$ anti-bonding orbital of D-X, (2) attractive electrostatic forces, (3) dispersion interaction, and (4) a repulsive term arising from Pauli exclusion principle. The weight of the above individual terms varies for different types of halogen bonding [17,18,19,20,21].

During the process of conceptualizing halogen bonding, theoretical and computational chemistry played an indispensable role. Politzer and co-workers first noticed the anisotropic electron density distribution around the donor halogen atom X [22] and they found in the elongation of D-X bond a small region with surprisingly positive electrostatic potential (ESP), which was then named σ-hole to explain the attraction between halogen atom X and nucleophile Y [23,24,25]. To understand the physical nature of the stabilization energy of halogen bonding, natural energy decomposition analysis (NEDA) [26] was employed by Infante and co-workers [27] to demonstrate that the σ-hole theory itself could not explain all halogen bonding situations and the contribution from $n\to \sigma^{*}$ charge transfer is substantial. Frontier orbital analysis [28] and natural bond orbital (NBO) analysis [29] were employed by Rosokha and co-workers to confirm the importance of charge transfer [30,31,32]. With symmetry-adapted perturbation theory (SAPT) [33,34], Stone came to similar conclusions that induction (which contains charge transfer) and dispersion are essential for halogen bonding besides the electrostatic term [35].

While many contributions have been made to obtain more accurate density functionals and more computationally attainable wavefunction theory methods for describing halogen bonding theoretically in terms of geometry and binding energy [17], the quest for theoretical tools emphasizing on bonding analysis is equally crucial for studying halogen bonding due to two reasons.

- The binding energy is a reaction parameter, summarizing all effects leading to bonding in a cumulative way. Even in a simple dimer the binding energy cannot serve as a measure for the intrinsic strength of a bond; it is contaminated with the stabilization energy of the two fragments caused by geometry relaxation and reorganization of the electron density of the fragments upon bond breakage [36]. This applies even more to complex systems with halogen bonding at work (e.g., a halogenated drug in a protein or halogen bonding in crystals);
- We need a bond strength measure that is not based on bond breaking and that follows Levine’s suggestion that chemistry is local [37].

Besides the above-mentioned analytical tools (e.g., NBO, SAPT), a few other tools have been employed in halogen bonding studies. The quantum theory of atoms in molecules (QTAIM) [38] can be employed to obtain the bond critical points (BCPs) of halogen bonding, showing that the electron density at BCPs correlates well with the interaction energy [39]. At (3, -1)-type BCPs, the local energy density by Cremer and Kraka determines whether a halogen bond is more covalent or ionic [40,41]. Methods like reduced density gradient (RDG) [42], electron localization function (ELF) [43], density overlap regions indicator (DORI) [44], and independent gradient model (IGM) [45] can identify the existence of halogen bonding graphically [46,47].

An important theoretical tool which has considerably contributed to a deeper understanding of halogen bonding is the *local vibrational mode theory* originally introduced by Konkoli and Cremer [48,49,50,51,52,53]. They derived local vibrational modes (associated with individual internal coordinates such as bond lengths, bond angles, etc.) directly from the normal vibrational modes (whose fundamental frequencies $\omega_{\mu }$ can be measured by infrared and Raman spectroscopy or calculated theoretically) by solving mass-decoupled Euler-Lagrange equations leading to a mass-decoupled analogue of Wilson’s equation of vibrational spectroscopy. Normal vibrational modes of polyatomic molecules are generally delocalized because of mass coupling [54,55,56,57] and therefore cannot directly be used as bond strength measure, which is an important fact but often overlooked.

Each local mode is associated with a corresponding local mode frequency $\omega_{n}^{a}$ and local mode force constant $k_{n}^{a}$. Zou and co-workers demonstrated that there is a one-to-one relationship between the local and normal vibrational modes that can be verified by an adiabatic connection scheme (ACS), providing a solid physical fundament for the local vibrational mode theory [52]. Zou and Cremer further proved that the local stretching force constant $k_{n}^{a}$ is directly related to the intrinsic strength of chemical bonds [58]. The underlying physical nature of this important proof results from the fact that $k_{n}^{a}$ equals the curvature of the potential energy surface (PES) in the direction of the bond stretching, determined via an infinitesimal change of the bond length and followed by the relaxation of all other atoms in the molecule [59,60]. In this way, $k_{n}^{a}$ absorbs all electronic effects contributing to an individual chemical bond and serves as unique measure of the intrinsic strength of a chemical bond and/or weak chemical interaction based on vibrational spectroscopy. In combination with other analytical tools (e.g., NBO analysis or electron density analysis) and the knowledge about well-studied systems, local stretching force constants offer a deeper and more comprehensive understanding of complex bonding situations, in particular determining whether the presence/absence of a specific electronic effect (e.g., π conjugation) is strengthening/weakening a chemical bond of interest in a comparative manner.

So far, the local mode analysis has been successfully applied to characterize covalent bonds [58,61,62,63,64,65,66] and weak chemical interactions such as intra- and inter-molecular hydrogen bonding in various forms and systems [67,68,69,70,71,72,73], chalcogen [74,75,76], pnicogen [77,78,79] and tetrel interactions [80], and in particular halogen bonding [81,82,83,84]. Recently, we extended the local vibrational mode theory from molecular to periodic one-dimensional (1D) through three-dimensional (3D) systems [85]. We consider it as an important step forward as it enables us to quantify and compare intrinsic bond strength in both materials/crystals and molecules, in particular considering (i) that currently only a few analytical tools are available for bonding analysis in periodic systems [86,87,88,89,90,91,92,93,94] due to the complication arising from the use of plane-wave basis set in first-principle modeling; (ii) the often reduced functionality of periodic versions of analysis tools originally designed for molecular systems (e.g., periodic NBO [95] does not provide interaction energies from second-order perturbation theory); and (iii) that the lattice structure intrinsically prohibits the calculation of bond dissociation energies.

In this work, we will apply our periodic local vibrational mode theory [85] to explore the X-I⋯OA-type halogen bonding (where X-I is the donor bond and I⋯O is the halogen bond) in molecular crystals in a systematic and comprehensive way. We chose this specific type of halogen bonding based on the following considerations.

1. The I⋯O halogen bonds account for a large portion of all halogen bonds ever discovered;
2. The oxygen acceptor atom is more common in molecular crystals than the higher chalcogens (e.g., S, Se and Te);
3. Dihalogen/interhalogen compounds X-I consist of only two atoms and therefore are the simplest halogen bond donors;
4. Recently, Rosokha and co-workers investigated the I-I⋯O-N-type halogen bonding in crystals with *N*-oxide acceptors via crystallography and theoretical calculations [96,97]. Their analysis based on molecular dimer models suggests that the charge transfer is a key factor in the I⋯O halogen bonding besides electrostatic attraction. Our work here based on a collection of crystal structures should provide a more detailed and comprehensive understanding of halogen bonding in materials.

The following objectives were pursued in this work.

- To create a comprehensive set of local stretching force constants for X-I⋯OA halogen bonds in different crystals describing the intrinsic halogen bond strength in these systems;
- To derive a more realistic model description considering the crystal packing effect explicitly and to understand factors that affect the solid state halogen bond strength by an in situ investigation of halogen bonding in a crystalline environment;
- To test the validity of the Badger’s rule [98,99,100], in particular the validity of the generalized Badger’s rule based on local stretching force constants [61] in crystals.

As the first systematic investigation of a series of non-covalent interactions using our periodic local vibrational mode theory, this paper will set an example for following projects, especially in terms of properly obtaining local mode force constants for periodic systems from first-principle calculations with sufficient accuracy.

The paper is structured in the following way: First, Computational Details are given. In the Results and Discussion section, the calculated crystal structures are discussed followed by a comparison of key structural features between computational and experimental results. Then we quantify the intrinsic strength of both donor and donor-acceptor bonds of X-I⋯O halogen bonding, as determined with periodic local vibrational mode theory. Important findings of this work are summarized in the Conclusions section and a future perspective is given.

## 2. Computational Details

All calculations in this work were carried out with the VASP 5.4.4 package [101,102,103,104,105] using van der Waals density functional rev-vdW-DF2 [106] with projector-augmented wave (PAW) potential [107,108]. The kinetic energy cutoff for basis set is 1000 eV. The rev-vdW-DF2 functional is a recently revised version of vdW-DF2 functional [109] with improved accuracy in describing van der Waals interactions (i.e., non-covalent bonding). According to a timely benchmark by Tran and co-workers, the rev-vdW-DF2 functional was shown to be the best choice for describing non-covalent interactions in molecular crystals [110].

All crystal systems investigated in this work were initially converted into primitive unit cells, as required by the definition of local vibrational modes in periodic systems [85]. The Monkhorst-Pack scheme [111] was used to sample the Brillouin zone with a k-point grid, where each lattice constant *a* times its number of k-points *k* is close to 28 Å, i.e., a·k∼28 Å. Noteworthy is that the settings for the energy cutoff and k-point sampling were based on delicate convergence testing of total energy and ionic forces. All halogen-bonded systems investigated in this work were optimized to local minima, verified via vibrational frequency calculations to ensure that there were no imaginary frequencies. A relatively tight criterion for geometry optimization as well as cell relaxation was adopted with the maximum ionic force less than 10${}^{-7}$ eV/Å. The force constant matrices were calculated numerically with analytic gradients using two displacements (±Δs) for each degree of freedom, where the step size Δs was set to 0.005 Å.

The molecules of diiodine (I${}_{2}$) and iodine monochloride (ICl) were calculated in a cubic box with the size of 20 Å and 24 Å, respectively, to simulate non-periodic calculations. Dipole corrections to the total energy were added for ICl along its dipole moment. Only the Γ point (k=0) was considered for sampling the Brillouin zone.

The intrinsic bond strength of the halogen bonds was quantified by their local stretching force constants $k_{n}^{a}$ derived from the local vibrational mode theory for periodic systems [85]. The periodic local mode analysis (whose time cost for local mode analysis in either molecules or crystals is comparable to normal mode analysis as long as the Hessian matrix has been calculated beforehand) was carried out with COLOGNE2019 package [112].

## 3. Results and Discussion

### 3.1. Selection of Molecular Crystals

This work was focused on the X-I⋯OA halogen bonding in crystals, of which the structures were retrieved from two major databases collecting crystal structures—the Cambridge Structural Database (CSD) [113] and the Crystallography Open Database (COD) [114] by performing searches with the keywords “dihalogen”, “interhalogen”, “diiodine” or their synonyms. 62 unique crystal structures with halogen bonding, matching the search keywords were obtained and then manually screened based on the following criteria:

- The molecular crystal should contain only the elements C, N, H, O/S/Se and X while excluding metal atoms;
- The total number of atoms in the primitive cell of the crystal should be preferably smaller than 80 to save computational cost;
- The dihalogen/interhalogen X-I should exist as neutral diatomic molecules instead of trihalogen cations.

20 crystal structures (as listed in the first column of Table 1) passed the screening procedure and they contain different dihalogen/interhalogen molecules including I${}_{2}$, IBr, ICl or Br${}_{2}$. To simplify the investigation, in particular with regard to a more consistent and straightforward analysis, we limited our investigation to I${}_{2}$ and ICl molecules with iodine atom as donor participating in the halogen bond. For the sake of enriching the data set, we carried out in silico crystal engineering by replacing the dihalogen/interhalogen molecules within those 20 crystals with either I${}_{2}$ or ICl molecules, leading to 20×2=40 model systems. The reasons why we chose I${}_{2}$ and ICl as the halogen bond donors are due to the following:

1. Iodine as the donor atom has relatively large polarizability and will more easily form a σ-hole than chlorine, bromine or fluorine;
2. Chlorine is more electronegative than bromine and it generally forms stronger X-I⋯O halogen bonds according to the σ-hole theory [23,24,25];
3. Although stronger halogen bonding is expected for iodine monofluoride (IF) as the donor molecule, this species is unstable and cannot form co-crystals under ambient conditions [115].

Furthermore, all sulfur and selenium atoms within the acceptor molecules of these 40 model systems were replaced with oxygens for consistency. These model systems were then used for VASP calculation to relax their lattice structures. The primitive cell structure of 33 model systems could be optimized while the remaining 7 systems either underwent drastic structural changes via isomerization or failed to meet the desired convergence criteria (see supplementary materials). It is worth noting that the overall arrangement of X-I donors and acceptor molecules in the optimized structures was retained compared to the original crystal structures without element replacement.

**Figure 1:**
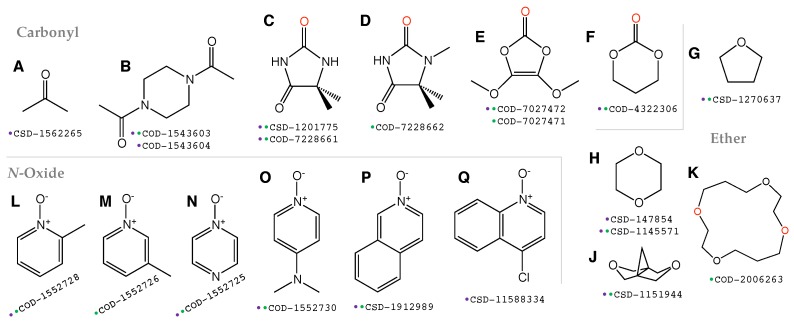
Structures of the 16 halogen bonding acceptor molecules investigated in this work. In any structure with two or more oxygen atoms, the oxygen participating in I⋯O halogen bonding is colored in red. Below each 2D molecular structure is the COD/CSD id number for the crystal structure(s) from which the acceptor molecule was extracted or adapted. The colored dot in front of the COD/CSD id number indicates that the model crystal structure containing the present acceptor molecule associated with diiodine (I${}_{2}$; purple dot) or iodine monochloride (ICl; green dot) was calculated in this work.

The calculations of the Hessian matrices confirmed that 32 model systems were optimized to local minima on the PES, while only one model system has a negative eigenvalue of its Hessian matrix and therefore was removed from our investigation. Subsequently, we obtained 34 unique X-I⋯O halogen bonds out of 32 model systems derived from the 20 crystal structures, as listed in Table 1. For a more systematic discussion, the 32 model systems were then organized according to the 16 acceptor molecules, shown in Figure 1, labeled **A**–**Q**.

**Table 1:** Summary of 34 X-I⋯O halogen bonding interactions investigated in this work.

ID ${}^{a}$	Label ${}^{b}$	$N_{\mathrm{atom}}$ ${}^{g}$	Donor Bond ${}^{f}$	*r* ${}^{c}$	$r_{\exp.}$ ${}^{d}$	$k_{n}^{a}$ ${}^{c}$	Halogen Bond *^e^*	*r* ${}^{c}$	$r_{\exp.}$ ${}^{d}$	$k_{n}^{a}$ ${}^{c}$
CSD-1562265 [116]	A	24	${}^{§}$I-I	2.7598		1.257	I⋯O=C(C${}_{2}$)	2.8133		0.087
	B1-1		I-I	2.7994		1.049		2.5281		0.309
COD-1543603 [117]	B1-2	30	Cl-I	2.4685	2.4142	1.252		2.3731	2.3864	0.575
COD-1543604 [117]	B2	56	${}^{§}$I-I	2.7753	2.7057	1.199	I⋯O=C(C,N)	2.7886	2.8195	0.143
CSD-1201775 * [118]	C1-1	38	I-I	2.7715		1.134		2.5170		0.370
	C1-2		Cl-I	2.4661		1.225		2.3888		0.588
	C2-1		I-I	2.7714		1.136		2.5195		0.368
COD-7228661 * [119]	C2-2	38	Cl-I	2.4661		1.224		2.3888		0.588
COD-7228662 * [119]	D	88	Cl-I	2.4447		1.335	I⋯O=C(N${}_{2}$)	2.4616		0.416
COD-7027472 * [120]	E1-1	36	I-I	2.7317		1.364		2.7340		0.149
	E1-2		Cl-I	2.4147		1.526		2.4958		0.412
COD-7027471 * [120]	E2	72	Cl-I	2.4207		1.513		2.5118		0.374
COD-4322306 * [121]	F-1	60	I-I	2.7667		1.146		2.6585		0.159
	F-2		Cl-I	2.4426		1.374	I⋯O=C(O${}_{2}$)	2.4469		0.461
CSD-1270637 * [122]	G-1	60	I-I	2.7724		1.176		2.5575		0.321
	G-2		Cl-I	2.4553		1.326		2.4145		0.561
CSD-147854 [123]	H1	16	${}^{§}$I-I	2.7685	2.6926	1.229		2.7565	2.8078	0.203
CSD-1145571 * [124]	H2-1	36	I-I	2.7718		1.115		2.6949		0.212
	H2-2		Cl-I	2.4277		1.454		2.5125		0.409
	J-1a			2.7790		1.090		2.6410		0.236
	J-1b		I-I	2.7745		1.123		2.6347		0.258
	J-2a			2.4493		1.330		2.4529		0.501
CSD-1151944 * [125]	J-2b	46	Cl-I	2.4263		1.477		2.4901		0.436
COD-2006263 * [126]	K	42	Cl-I	2.4319		1.415	I⋯O(C${}_{2}$)	2.5616		0.341
COD-1552728 [97]	L-1	68	I-I	2.8168	2.7509	0.999		2.4488	2.4803	0.413
	L-2		Cl-I	2.5022		1.120		2.3281		0.716
COD-1552726 [97]	M	34	Cl-I	2.4930		1.114		2.3373		0.664
COD-1552725 [97]	N-1	26	${}^{§}$I-I	2.8073	2.7340	1.036		3.1606	3.0345	0.164
	N-2		Cl-I	2.4462		1.361		2.4151		0.489
	O-1		I-I	2.8629	2.7952	0.841		2.3857	2.3587	0.565
COD-1552730 [97]	O-2	44	Cl-I	2.5446		0.940		2.3030		0.783
CSD-1912989 [97]	P-1	40	I-I	2.8188	2.7512	0.966		2.4470	2.4637	0.435
	P-2		Cl-I	2.5096		1.059		2.3327		0.681
CSD-1588334 [96]	Q	40	I-I	2.8118	2.7328	1.003	I⋯O-N(C${}_{2}$)	2.4850	2.4990	0.198
Diiodine	I${}_{2}$	2	I-I	2.6919	2.6660	1.667				
Iodine monochloride	ICl	2	Cl-I	2.3413	2.3207	2.233				

${}^{a}$ The id number of the crystal structure from COD/CSD database. The “*” symbol indicates that sulfur, selenium or tellurium atoms have been replaced with oxygen atoms in this crystal structure. ${}^{b}$ The label for each halogen bond is formatted as X i-ii y, where letter “X” denotes a specific acceptor molecule as shown in Figure 1, number “i” denotes the first or second COD/CSD structure, number “ii” denotes whether the donor molecule is diiodine or iodine monochloride and letter “y” denotes the first or second halogen bonding in the same crystal model system. ${}^{c}$ Calculated bond length *r* and local stretching force constant $k_{n}^{a}$ in the unit of Angstrom (Å) and mdyn/Å, respectively. ${}^{d}$ Experimentally measured bond length $r_{exp.}$ with the unit of Angstrom (Å) in crystal structures. *^e^* This table is divided into 7 sections based on the halogen bond type. The shade in gray color is used solely for distinguishing different COD/CSD structures within each section. ${}^{f}$ The symbol *§* before a X-I donor means there exists a close contact (i.e., a halogen bond) with a distance of less than 3.0 Å with regard to the atom X. ${}^{g}$ Total number of atoms in the primitive cell model. The local mode frequencies $\omega_{n}^{a}$ for I-I bond stretching can be calculated by $\omega_{n}^{a}=\sqrt{k_{n}^{a}}\cdot 163.6$, where $k_{n}^{a}$ is in the unit of mdyn/Å and the resulting vibrational frequency $\omega_{n}^{a}$ is in the unit of cm${}^{-1}$. The local mode frequencies $\omega_{n}^{a}$ for Cl-I bond stretching can be calculated by $\omega_{n}^{a}=\sqrt{k_{n}^{a}}\cdot 248.8$, where $k_{n}^{a}$ is in the unit of mdyn/Å and the resulting vibrational frequency $\omega_{n}^{a}$ is in the unit of cm${}^{-1}$. The local mode frequencies $\omega_{n}^{a}$ for I⋯O bond stretching can be calculated by $\omega_{n}^{a}=\sqrt{k_{n}^{a}}\cdot 345.5$, where $k_{n}^{a}$ is in the unit of mdyn/Å and the resulting vibrational frequency $\omega_{n}^{a}$ is in the unit of cm${}^{-1}$.

### 3.2. Comparison of Experimental and Calculated Structures

**Figure 2:**
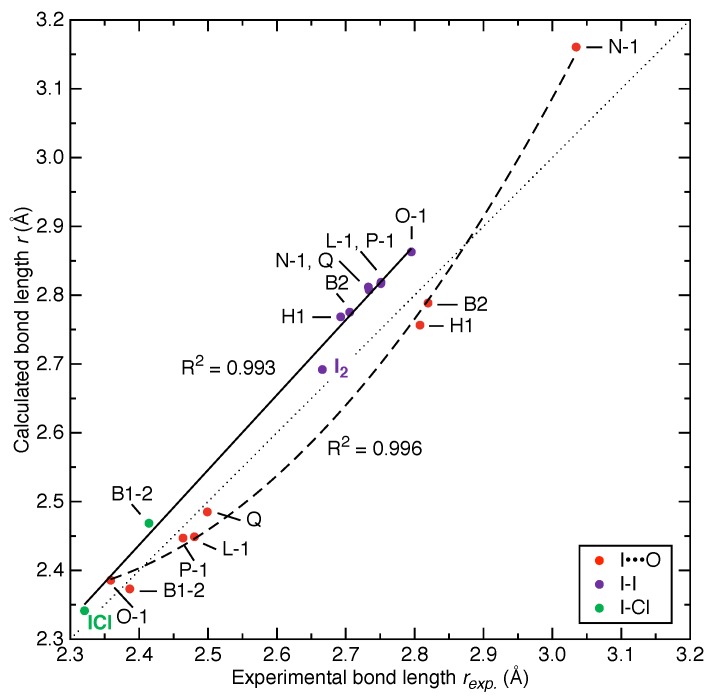
Comparison between calculated versus the experimental bond lengths in 8 crystal structures. The diagonal dotted line is y=x, i.e., reflecting 100% agreement between experimental and calculated values. Purple and green dots represent I-I and I-Cl covalent bonds, respectively. The diiodine (I${}_{2}$) and iodine monochloride (ICl) molecules in gas phase are also included for comparison. The solid black line shows a linear fit ($R^{2}=0.993$) for I-I and I-Cl covalent bonds altogether. The red dots representing I⋯O halogen bonds are best fitted with a quadratic curve ($R^{2}=0.996$) shown as the dashed black line.

In contrast to analytical tools qualitatively describing chemical bonding from properties directly extracted from experimentally resolved crystal structures (e.g., the Hirshfeld charge/surface analysis [127]), our local vibrational mode analysis requires the system of interest to be optimized into a local minimum point on the PES. Therefore, it becomes necessary to check the deviation of the optimized structure from the single-crystal X-ray structure. In Figure 2, the bond lengths for both the X-I donor bonds and I⋯O halogen bonds of the X-I⋯OA halogen bonding in 8 crystal structures (as listed in Table 1) are examined.

The calculated covalent bond lengths of I${}_{2}$ and ICl molecules in these 8 systems are slightly longer than those extracted from the experimental structures. We also considered the isolated I${}_{2}$ and ICl molecules in gas phase calculated with the same level of theory and their calculated bond lengths are also marginally longer than experimental values [115]. We combined the I-I and I-Cl distances together and observed a strong linear correlation with a coefficient of determination ($R^{2}$) as 0.993 between the calculated and experimental bond lengths. On the other hand, most of the bond lengths for I⋯O interactions are slightly underestimated but they seem to follow a quadratic function with $R^{2}=0.996$ between the calculated and measured values, which is surprising. To the best of our knowledge, it is the first time that one has observed a linear correlation for donor bonds along with a quadratic correlation for donor-acceptor bonds for a certain type of non-covalent interaction between calculated and experimentally measured bond lengths. We postulate that the quadratic correlation for I⋯O interactions is the result of a second-order perturbation while the covalent X-I bonds follow a first-order perturbation. Further validation of such relationships for other types of donor-acceptor interactions is currently under investigation. This interesting bond length-bond strength relationship can be potentially useful to predict key parameters in the experimental crystal structure based on a calculated model or as a novel metric to benchmark the quality of density functionals [128].

### 3.3. Intrinsic Strength of Donor Bonds and Halogen Bonds in X-I⋯OA Halogen Bonding

#### 3.3.1. General Trends

**Figure 3:**
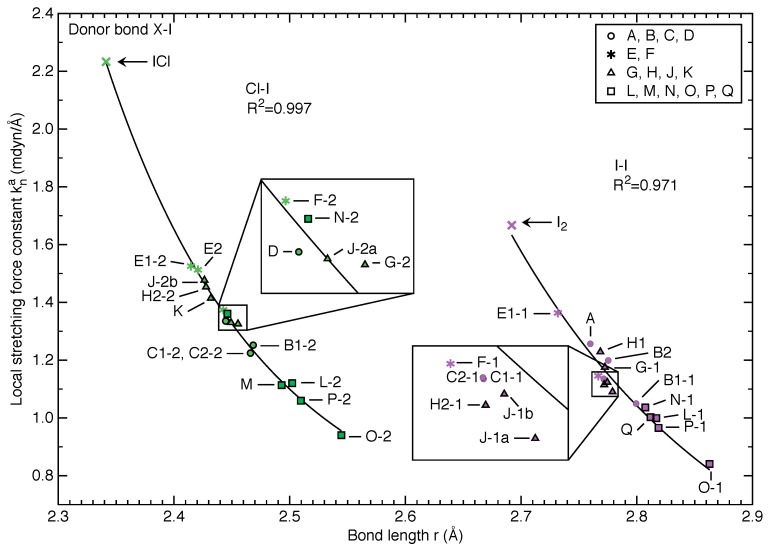
Relationship between local stretching force constant $k_{n}^{a}$ and bond length *r* for Cl-I (green) and I-I (purple) covalent bonds, respectively. Data points are shown with at least 4 shapes based on the acceptor molecule type, see Figure 1. Power functions in the form of $k_{n}^{a}=a\cdot r^{b}+c$ were employed in fitting the data points for Cl-I and I-I bonds separately.

**Figure 4:**
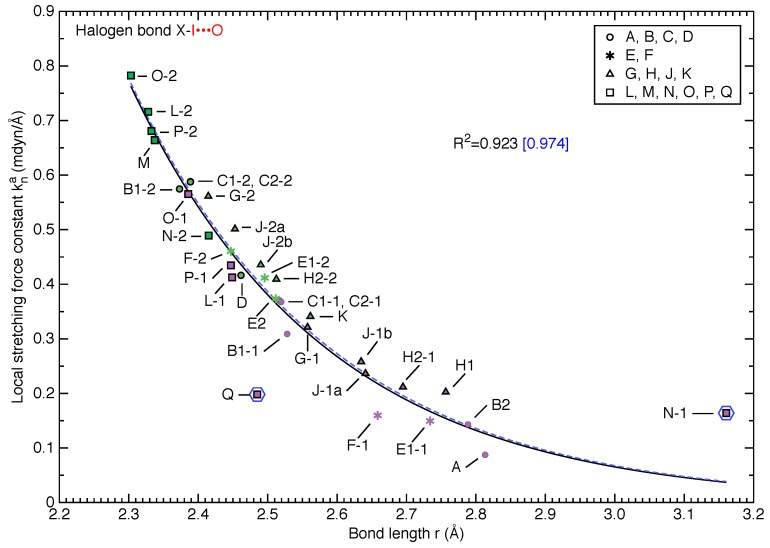
Relationship between local stretching force constant $k_{n}^{a}$ and bond length *r* for Cl-I⋯O (green) and I-I⋯O (purple) halogen bonds, respectively. Data points are shown with 4 shapes based on the acceptor molecule type, see Figure 1. An exponential function in the form of $k_{n}^{a}=a\cdot exp\left(b\cdot r\right)$ was used to fit all 34 data points with $R^{2}=0.923$. Two data points are identified as outliers (encircled by blue hexagons) and $R^{2}=0.974$ is obtained for fitting the remaining 32 data points. The blue dashed curve shows the updated fitting function excluding two outliers.

Figure 3 and Figure 4 show the relationship between local stretching force constant $k_{n}^{a}$ and bond length *r* for 34 halogen bonding scenarios in terms of X-I donor bond and I⋯O halogen bond, respectively. More detailed information for these halogen bonds is collected in Table 1. As revealed in Figure 3, we find a strong correlation between force constant and bond length for I-I donor bonds ($R^{2}=0.971$) and an even stronger correlation ($R^{2}=0.997$) for the Cl-I donor bonds. The correlation between force constant and bond length for the I⋯O bonds shown in Figure 4 is somewhat weaker with $R^{2}=0.923$ and $R^{2}=0.974$ by excluding two outliers. This reveals that in essence the Badger’s rule [98,99,100] still holds, namely shorter bonds have larger harmonic frequencies (or force constants) and are thus stronger. Given that the Badger’s rule was generalized to polyatomic molecules based on local stretching force constant by one of the authors of this work [61] and was recently extended to O-H bonds in liquid water [129], this work demonstrates for the first time that the Badger’s rule even holds for crystals.

Figure 3 shows that all Cl-I covalent bonds are shorter than I-I bonds by at least 0.15 Å and the Cl-I bonds in crystals have generally larger local stretching force constant values than I-I bonds although they overlap in the range of 0.95–1.35 mdyn/Å. All X-I covalent bonds in crystal models are longer and weaker than their molecular counterparts in gas phase, which is a result of delocalization of lone pair electrons into the $\sigma^{*}$(X-I) anti-bonding orbital [97] upon halogen bonding in crystals. Furthermore, we found that when the halogen bond acceptor is an ether group (**G**, **H**, **J**, **K**) the X-I bond is stronger than those when halogen bonds have *N*-oxides (**L**, **M**, **N**, **O**, **P**, **Q**) as acceptors.

It is worth noting that two distinct local stretching force constant-bond length relationships were observed for Cl-I and I-I bonds separately in Figure 3. This result is in line with our previous work where the essential difference between Badger-type rules for diatomic molecules and those for polyatomic compounds lies in that in the case of polyatomic molecules an individual curve can be expected for each bond type [61]. So, the important finding of this work is that rules worked out for the description of molecules in the gas phase seem to smoothly transition from bonding in molecules towards bonding in crystals.

Figure 4 collects two types of halogen bonds as Cl-I⋯O and I-I⋯O. Although the X atom in X-I donor bond is different, two halogen bonding atoms (i.e., I and O) are consistent and therefore the local stretching force constants for these two types of I⋯O bonds can be directly compared to provide detailed chemical insights. Such legitimacy for a direct comparison was also reflected by the uniformly strong correlation between force constants and bond lengths for these two types of halogen bonds.

We find that the Cl-I⋯O halogen bonds are generally shorter and stronger than I-I⋯O bonds, which is in line with the σ-hole theory [23,24,25]. Within either type of halogen bond, the intrinsic strength is mostly larger when the acceptor is *N*-oxide (**L**, **M**, **N**, **O**, **P**, **Q**) than those halogen bonds with ether group (**G**, **H**, **J**, **K**) as acceptors. This can be explained in terms of the charge transfer character (i.e., orbital interaction) [97] that the oxygen atom in *N*-oxide group could provide more lone pair electrons to be delocalized into the $\sigma^{*}$(X-I) anti-bonding orbital for halogen bonding than the oxygen in an ether group.

#### 3.3.2. Acceptor **A–F**

The acceptors **A** through **F** all provide a carbonyl C=O double bond to accept halogen bonding where **A** is acetone, **B** has two equivalent carboxamide groups, **C** and **D** are urea derivatives while **E** and **F** have carbonate ester groups.

Figure 3 shows that the X-I donor bonds in halogen bonding E1-1, E1-2 and E2 with acceptor **E** are the strongest for the crystal models studied in this work, although they are weaker than their molecular counterparts. This scenario can be explained in the following way. The intrinsic strength of a donor X-I bond is largely influenced by the extent to which the lone pair electrons of acceptor oxygen delocalize into the $\sigma^{*}$ anti-bonding X-I orbital. In addition, acceptor **E** has a delocalized π electron cloud throughout the whole plane and two strongly electronegative oxygen atoms distant from the C=O double bond can “pull” the oxygen lone pair charge density around the double bond towards the carbon atom. This polarization leads to a stronger C=O bond, as shown in Table 2 making the C=O bond of acceptor **E** the strongest among acceptors **A** through **F**, obviously suppressing the charge transfer character to the neighboring halogen bond. This is in line with Figure 4 showing that halogen bonds E1-1, E1-2 and E2 are relatively weak for either X-I⋯O type.

**Figure 5:**
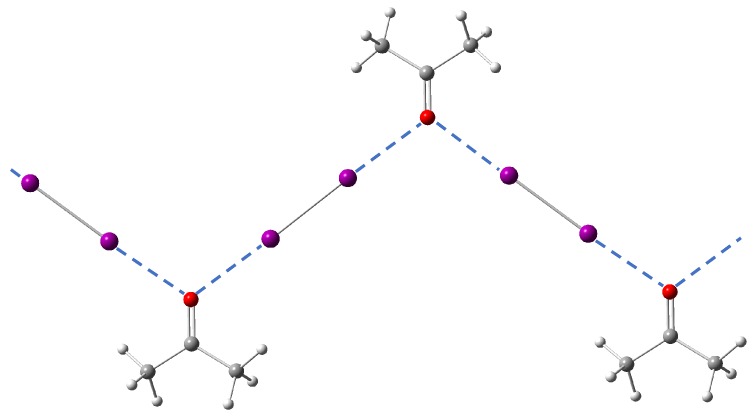
Optimized crystal model structure showing the bifurcated halogen bonding A between diiodine and acceptor molecule **A**.

The I⋯O bond of halogen bonding A is the weakest among all halogen bonds studied in this work, whereas its donor I-I bond is relatively strong. By checking the corresponding crystal structure of A (see Figure 5), we found that both sides of each I${}_{2}$ molecule form I⋯O halogen bonds and the oxygen atom of each acetone molecule accepts two I⋯O halogen bonds at the same time. In this situation, the lone pair electrons from one oxygen atom have to be shared by two donor I${}_{2}$ molecules and the charge transfer into either I${}_{2}$ donor is greatly reduced compared with the unshared situation (based on the physical picture of NBOs and their interaction), thus leading to weak halogen bonds and strong donor bonds. As the overall charge transfer from the acetone molecule is weak, the charge density of the C=O double bond region is less polarized towards oxygen atom and therefore leads to strong C=O bonds as shown in Table 2. This clearly shows that the crystal environment plays an important role which can be sensitively reflected by the local stretching force constants.

The halogen bond B2 has the second lowest intrinsic strength as shown in Figure 4. The optimized crystal model for B2 reveals that each I${}_{2}$ molecule is connected to two carboxamide groups, acting as a linear bridge connecting two C=O groups with equal I⋯O distances. This unique structural arrangement weakens the I⋯O halogen bond because of the following. First, the two iodine atoms of I${}_{2}$ are symmetrically equivalent in terms of charge density, which reduces the area of the σ-holes on both sides. Second, the lone pair electrons from the oxygen atoms on both sides of I${}_{2}$ compete with each other in delocalizing into the $\sigma^{*}$ anti-bonding orbital of I${}_{2}$, leading to reduced charge transfer for both I⋯O bonds. This explanation is rationalized by the fact that the C=O double bond of acceptor **B** is strongest in the case of B2 halogen bonding compared with B1-1 and B1-2 (see Table 2) as the I${}_{2}$/ICl molecules in B1-1/B1-2 form only one I⋯O halogen bond.

#### 3.3.3. Acceptor **G–K**

The molecules **G** through **K** possess ether groups as halogen bond acceptors. For the associated halogen bonds of either X-I⋯O type, we found a consistent strength ordering according to the acceptor type as **G**>**J**>**H**(>**K**) although their overall I⋯O bond strength is weak.

Such a consistent ranking can be explained by a through-bond interaction between oxygen atoms in these four acceptor molecules. The tetrahydrofuran acceptor **G** has only one oxygen atom, thus the charge transfer from this oxygen is not affected. In acceptor **J**, two acceptor oxygen atoms start to suppress the charge transfer of each other via their high electronegativity although they are relatively distant. The 1,4-dioxane acceptor **H** molecule has also two oxygen atoms to accept halogen bonding, but they are on *para* positions of a six-membered ring and much closer to each other than in molecule **J**. Therefore, the charge transfer from the oxygens in **H** is more suppressed than in **J**. **K** has four oxygen atoms with only two of them participating in halogen bonding in crystals. Due to the large number of oxygen atoms, the charge transfer from oxygen to halogen bonding is most strongly suppressed in **K**.

#### 3.3.4. Acceptor **L–Q**

The acceptor molecules **L** through **Q** are heteroaromatic *N*-oxides, where the N^+^-O^−^ bond is linked to an aromatic ring [97].

We find that the donor X-I bonds associated with acceptor molecule **O** (O-1 and O-2) are the weakest, while the corresponding I⋯O bonds are the strongest among either X-I⋯O group. These findings can be rationalized in the following way. The nitrogen and oxygen atoms in an N^+^-O^−^ bond have comparable electronegativity, but oxygen attracts slightly more bonding electron density. One also needs to consider the substituent effects of the -N(CH${}_{3}$)${}_{2}$ amine group linked to an aromatic ring. As it is well-known that amine group has an *ortho*-/*para*-directing effect for electrophilic aromatic substitution reactions [130,131,132,133], so the nitrogen atom of the N^+^-O^−^ bond in the *para* position with regard to the amine group in acceptor **O** can attract more electron density from the aromatic ring while loosening the attraction of the NO bonding electrons. Therefore, the oxygen atom of the N^+^-O^−^ bond can accumulate electron density via its large electronegativity. This leads to the weakest N^+^-O^−^ bond for acceptor **O** as shown in Table 3. The charge transfer from the oxygen lone pair electrons into the donor I${}_{2}$/ICl molecule is greatly enhanced resulting in strong halogen bonds with weak donor bonds.

Another interesting observation is that N-1 and N-2 halogen bonds are the weakest for either X-I⋯O type when acceptors are *N*-oxides. This is mainly caused by the electronic structure of acceptor molecule **N**. First, molecule **N** has a pyridine-like structure as shown in Figure 1. The nitrogen atom in a pyridine molecule acts like a *meta*-directing group for electrophilic aromatic substitution reaction [132], similarly the nitrogen atom (position 4) within acceptor molecule **N** makes the other nitrogen atom (position 1) of N^+^-O^−^ bond slightly electron-deficient. This helps pulling the bonding electrons of the N^+^-O^−^ bond towards the nitrogen atom making this N^+^-O^−^ bond stronger. This is in line with the fact that the N^+^-O^−^ bonds within the acceptor molecule **N** are the strongest in Table 3. Then one can infer that the charge transfer from the oxygen atom will be reduced and therefore the corresponding halogen bond is weakened.

The substituent effect in acceptor molecules **L**, **M** and **P** on their N^+^-O^−^ bonds is more similar to that in acceptor molecule **O** than in acceptor molecule **N**, as revealed by corresponding local stretching force constants being larger but close to the force constant values for acceptor molecule **O** (see Table 3). This explains why the local stretching force constants of I⋯O bonds associated with these three acceptor molecules are between those of I⋯O bonds associated with acceptor molecule **O** and acceptor molecule **N** within either X-I⋯O type.

This work includes five I-I⋯O halogen bonds overlapping the recent work by Rosokha and co-workers [96,97] including P-1(iQnO), L-1(2MePyO), O-1(Me2NPyO), N-1(PyrazO) and Q(ClQnO) (abbreviations in parentheses are taken from Reference [97]). The ordering for intrinsic strength of the first four halogen bonds (see Figure 4) is consistent with the ordering for binding energies of dimer complexes, except for P-1 and L-1. However, these two halogen bonds have a binding energy difference of only 0.2 kcal/mol [97] in line with our local mode analysis also showing marginal difference in local stretching force constant of 0.033 mdyn/Å. This means the binding energies calculated from dimer models with different acceptors qualitatively predict the strength of the halogen bonds in these four crystals. However, the I⋯O bond of halogen bonding Q is much weaker than expected although corresponding binding energy between diiodine and acceptor **Q** in a dimer complex is comparable to those for acceptors **L** and **P** according to Rosokha’s calculations [97]. Moreover, the local stretching force constant of the N^+^-O^−^ bond in acceptor **Q** indicates that this halogen bond is likely to have comparable strength as P-1, as shown in Table 3.

#### 3.3.5. Outliers

Figure 3 showing the relationship between local stretching force constant and bond length for donor X-I bonds has no obvious outliers deviated from the fitted curves. However, there are a few outliers around the fitted curve for I⋯O bonds in Figure 4. Besides Q and N-1 identified as two outliers by the deviation criterion of 1.5·σ (σ is the standard deviation of the residuals after fitting), we found halogen bonds A, H1 and F-1 also have relatively large deviation from the best fitted curve.

In the case of halogen bonding A, the I⋯O bond force constant is lower than what the fitted curve predicts by 0.05 mdyn/Å. As pointed out in Section 3.3.2, in its crystal structure with acetone as the acceptor molecule each C=O double bond accepts two halogen bonds instead of one (see Figure 5). This is the major reason halogen bond A becomes an outlier given its peculiar bifurcated halogen bonding geometry.

In the optimized crystal structure of halogen bond F-1, one iodine atom of I${}_{2}$ forms the F-1 halogen bond, while the other side of the I${}_{2}$ molecule is pointing towards an oxygen atom on the six-membered ring of another acceptor molecule **F** with the I⋯O distance of 3.82 Å. As the I-I bond is nearly perpendicular to the plane of this distant carbonate ester group which is a π conjugated system, the charge transfer into I${}_{2}$ might be non-trivial given the large radius of the valence shell of iodine atom. Moreover, the electron-rich carbonate ester group with three oxygen atoms could be an ideal halogen bond acceptor to form the second halogen bonding with I${}_{2}$. This explains why halogen bond F-1 is weaker than what can be expected from the fitted curve, because the other side of the I${}_{2}$ molecule is affected by another seemingly distant acceptor molecule. One might doubt why the halogen bond F-2 is not affected to be an outlier, we argue that the chlorine atom of the donor Cl-I bond in F-2 is not a good halogen bond donor according to the σ-hole theory and the charge transfer from oxygen towards chlorine is more difficult given the Cl⋯O distance as 3.92 Å. The halogen bond F-2 experiences little influence from the other side of ICl donor and therefore its I⋯O bond is not an outlier.

**Figure 6:**
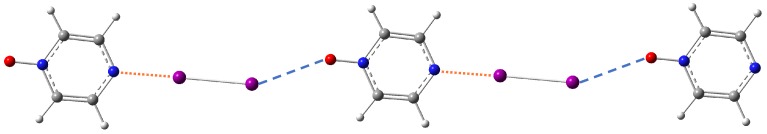
Optimized crystal model structure showing the I⋯O halogen bonding N-1 (blue dashed lines) between diiodine and acceptor molecule **N** within the infinite alternating chain. The I⋯N halogen bonding is shown with orange dotted lines.

For halogen bond N-1 identified as an outstanding outlier in Figure 4, the other side of each I${}_{2}$ molecule forms a rather short (maybe stronger) I⋯N halogen bonding with another acceptor molecule **N** (see Figure 6). This I⋯N interaction strongly outweighs the I⋯O bonding and makes N-1 an outlier in Figure 4. We notice that the local stretching force constant of the N-1 halogen bond is larger than what the fitted curve predicts by more than 0.1 mdyn/Å. This strengthening is caused by the strong polarization of the push-pull effect [71] (which enhances the charge transfer from oxygen to I${}_{2}$ molecule) arising from the infinite alternating chain of I${}_{2}$ and acceptor molecule **N** [97].

**Figure 7:**
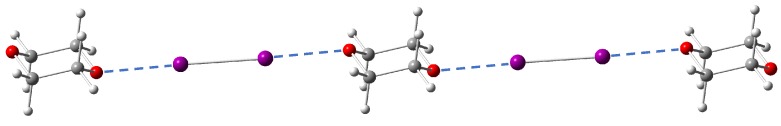
Optimized crystal model structure showing the halogen bonding H1 between diiodine and acceptor molecule **H** within the infinite alternating chain.

In the optimized crystal structure for halogen bond H1, we also found an infinite alternating chain consisting of I${}_{2}$ and 1,4-dioxane acceptor **H**. As shown in Figure 7, each oxygen atom of the dioxane ring forms a halogen bond with an identical I⋯O distance and an I${}_{2}$ molecule donates two equivalent halogen bonds connecting two dioxane rings. We believe this infinite chain structure might render H1 as an outlier above the fitting curve. However, there is no push-pull effect [71] in this case, because both I${}_{2}$ and acceptor molecule **H** have no dipole moments within the infinite chain structure. The unusual strengthening for H1 halogen bond is explained as follows. We first consider a ternary segment of **H**⋯I${}_{2}\cdots$**H** within the infinite chain. Two oxygen atoms within molecules **H** on both sides of this ternary complex tend to attract electrons caused by their strong electronegativity. This through-bond interaction suppresses the charge transfer from two inner oxygens into the central I${}_{2}$ molecule. After we put this ternary segment back into the infinite chain structure, those two flanking oxygens in that ternary complex also form halogen bonding and their suppressive effect on the said charge transfer is reduced. Therefore, the I⋯O bond in H1 is strengthened by the infinite alternating chain.

One might raise the question why the halogen bond B2 is not an outlier although it has similar infinite alternating chain structure as H1 without push-pull effect. Based on the above analysis for H1, this might be related to the fact that two oxygen atoms of carboxamide groups within acceptor **B** are more distant than in acceptor **H**. In this way, the halogen bonding within a ternary model **B**⋯I${}_{2}\cdots$**B** is similar to halogen bonding B2 in crystals (with infinite alternating chain) due to minor influences from the two flanking carboxamide groups in the ternary complex.

**Figure 8:**
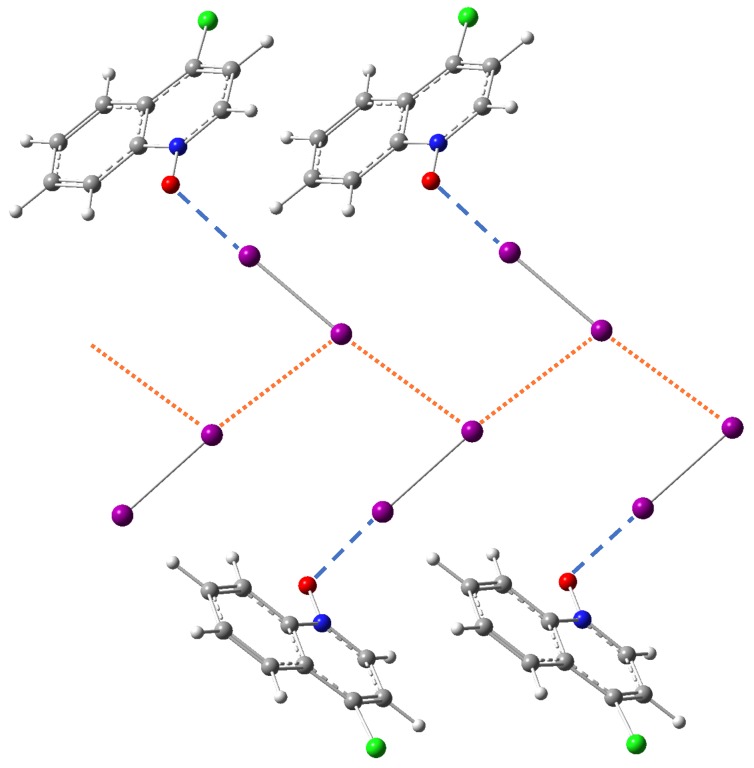
Optimized crystal model structure showing the I⋯O halogen bonding Q (blue dashed lines) between diiodine and acceptor molecule **Q**. The orange dotted lines show the I${}_{2}\cdots$I${}_{2}$ interaction network.

The I⋯O bond of halogen bonding Q is the most significant outlier in Figure 4. Unlike other outliers discussed above, it has a much shorter bond length of ca. 2.5 Å, indicating that there might be a different mechanism making it an outlier. By checking the crystal structure containing halogen bond Q, we found there exists a highly ordered I${}_{2}$ network between two rows of acceptor molecules **Q** (see Figure 8). For each halogen bonding Q, the other side of donor I${}_{2}$ forms two I⋯I halogen bonds of equal distance, and the resulting zigzag pattern is repeated as an infinitely long one-dimensional network [96]. These peculiar interactions between diiodine molecules are the major factor which greatly weakens the I⋯O bonds. Similar interaction networks between diiodines were also found in recent work on metal-organic framework (MOF) structures designed for I${}_{2}$ adsorption [134]. A more detailed and systematic investigation into this problem is currently in progress.

#### 3.3.6. Crystal Packing Effect

By taking a bird’s eye view of Figure 4, we found that when the donor X-I bond forms the second halogen bond with a distance less than 3.0 Å as in the case of the I⋯O bonds A, B2, H1 and N-1 (as listed in Table 1), these halogen bonds have relatively small local stretching force constants equal or less than 0.2 mdyn/Å. Furthermore, these are the only halogen bonds with a bond length larger than 2.75 Å. Moreover, halogen bonds Q and F-1 are also characterized by small local stretching force constants less than 0.2 mdyn/Å. The above six halogen bonds have one thing in common, their donor I${}_{2}$ molecule forms a second halogen bond. The local stretching force constant less than 0.2 mdyn/Å for the halogen bond E1-1 is caused by the strong substituent effect in acceptor molecule **E** (as discussed in Section 3.3.2).

Removing all data points with local stretching force constants less than 0.2 mdyn/Å from Figure 4 except E1-1, we found all remaining data points stay quite close to the fitted curve between bond length and local stretching force constant, rigorously following the Badger’s rule. In addition, we found all Cl-I⋯O halogen bonds stay close to the fitted curve because the chlorine atom is not a good donor to form a second halogen bond. This means that the impact from crystal packing on the I⋯O halogen bonding will be non-trivial if the donor I-I bond forms a second halogen bonding with oxygen, nitrogen or diiodine.

However, the causal relationship between the weak I⋯O bond, and the existence of the second halogen bond arising from crystal packing is complicated and it may vary in different situations:

- In the case of halogen bonding Q, reference DFT calculations on dimer models by Rosokha and co-workers have demonstrated that the acceptor molecule **Q** and diiodine could form a halogen bond as strong as the halogen bond between acceptor molecule **P** and diiodine [97]. However, in the crystal structure, the I${}_{2}\cdots$I${}_{2}$ interaction network dominates the crystal packing and it directly leads to weaker I⋯O halogen bonds than expected;
- In the case of halogen bonding N-1, the I⋯O halogen bond is weak because the molecule **N** is a poor acceptor. In this case, the crystal packing must enforce the structure to form a second halogen bonding for compensation to stabilize the whole system.

These subtle effects cannot be considered by calculations on dimer models, showing the necessity of in situ quantification of chemical bond strength in crystal structures.

## 4. Conclusions

In this work, we accurately quantified and systematically ordered the intrinsic strength of 34 X-I⋯OA halogen bonds (where X=Cl or I) from 20 different crystal structures by employing our periodic local vibrational mode theory [85]. The most important findings include the following:

1. We observed strong correlations between bond length and local stretching force constant for both X-I donor bonds (i.e., I-I and Cl-I) and I⋯O halogen bonds, which suggests that the generalized Badger’s rule (based on local stretching force constants [61]) originally derived from molecules is also valid for both covalent bonds and non-covalent interactions in crystals;
2. The local stretching force constants for I⋯O halogen bonds (Figure 4) span a wide range of 0.1–0.8 mdyn/Å, demonstrating the impressive tunability in bond strength even within a specific type of halogen bonding;
3. Our results for some I-I⋯O halogen-bonded crystals previously investigated experimentally and computationally by Rosokha and co-workers [96,97] clearly show the potential of the periodic local mode analysis leading to new deeper insights:
  (a) Rosokha’s statement that “besides electrostatic, molecular orbital interactions play a substantial role in XB between diiodine and *N*-oxides” can be expanded to the I⋯O halogen bonding between dihalogen X-I and any acceptor oxygen atom with lone pair electrons. This generalization is based on the strong correlations between bond length and force constant for both donor bonds and halogen bonds in Figure 3 and Figure 4;
  (b) In comparison to the dimer model systems used for DFT calculations by Rosokha and co-workers, our first-principle calculations on crystal models could include the crystal packing effects. On one hand, the overall lattice structure (including donor/halogen bond lengths) of molecular crystals is a direct result of crystal packing. On the other hand, the impact from crystal packing on X-I⋯OA halogen bonding varies in different ways:
    - In those cases where the X atom of the X-I halogen donor molecule has no close contact to neighboring atoms in the crystal or is only stabilized by an interaction with the π cloud of an adjacent aromatic ring, the I⋯O halogen bonds behave like covalent bonds and rigorously follow a local stretching force constant-bond length relationship;
    - When both sides of X-I donor are involved in halogen bonding (only observed for I${}_{2}$ not for Cl-I), the I⋯O halogen bond is weak. If the acceptor oxygen atom has to accept two halogen bonds simultaneously (e.g., halogen bonding A), the halogen bond strength is even lower. If the X atom forms a non-trivial halogen bond which obscures the target I⋯O halogen bond (e.g., N-1 and Q), the target halogen bond largely deviates from the ideal force constant-bond length relationship.
    - However, independent of the engagement of the X atom in additional halogen bonding associated with crystal packing, the donor bonds rigorously follow an ideal force constant-bond length relationship (Figure 3) due to their covalent bond nature.
  (c) Via delicate analysis in terms of substituent effect and other electronic structure factors in acceptor molecules, we are able to explain the majority of the variations in the intrinsic strength of both donor bonds and halogen bonds in X-I⋯OA bonding. Furthermore, the local stretching force constants of the adjacent O-A bonds in acceptor molecules could complement our findings;
  (d) In determining the strength of I⋯O halogen bonds, halogen atom X within X-I donor plays a decisive role as the weakest Cl-I⋯O halogen bonds are comparable to the strongest I-I⋯O halogen bonds. The acceptor molecules with different capabilities for $n\to \sigma^{*}$(X-I) charge transfer are the second important factor for determining the I⋯O bond strength. Last but not least, the existence of the second halogen bonding via the X atom of the donor X-I bond can substantially weaken the target I⋯O halogen bond in crystals.
4. We discovered for the first time a linear correlation for X-I donor bonds along with a quadratic correlation for I⋯O halogen bonds between experimental and calculated bond lengths. One application based on this is to estimate the local stretching force constant of either the X-I donor bond or I⋯O halogen bond of X-I⋯OA halogen bonding directly for a newly resolved crystal structure via the correlations in Figure 2, Figure 3 and Figure 4 given that no second halogen bond exists with atom X of the X-I donor molecule. Such relationship may also hold for other types of non-covalent interactions;
5. All calculations in this work were based on projector-augmented wave (PAW) basis. The resulting chemically sound results of local stretching force constants demonstrate that our periodic local mode analysis is not limited to atomic orbital (AO)-based computational results [85], it is generally applicable and independent of how the wavefunction is obtained. Using periodic local vibrational mode theory as a unique tool to investigate the intrinsic strength of other types of halogen bonding (and non-covalent interactions) in crystals/materials will be one of our current and future directions.

## Figures and Tables

**Table 2:** Local stretching force constants of the C=O bonds for acceptor molecules **A–F** in descending order.

No.	I-I⋯O=C	$k_{n}^{a}$ (mdyn/Å)	Cl-I⋯O=C	$k_{n}^{a}$ (mdyn/Å)
1	E1-1	10.327	E1-2	9.932
2	A	9.755	E2	9.905
3	F-1	9.000	F-2	8.807
4	C1-1	8.945	C1-2	8.579
5	C2-1	8.937	C2-2	8.575
6	B2	8.431	D	8.561
7	B1-1	7.917	B1-2	7.651

The local mode frequencies $\omega_{n}^{a}$ for C=O bond stretching can be calculated by $\omega_{n}^{a}=\sqrt{k_{n}^{a}}\cdot 497.6$, where $k_{n}^{a}$ is in the unit of mdyn/Å and the resulting vibrational frequency $\omega_{n}^{a}$ is in the unit of cm${}^{-1}$.

**Table 3:** Local stretching force constants of the N^+^-O^−^ bonds within acceptor molecules **L–Q** in ascending order.

	I-I⋯O^−^-N^+^	$k_{n}^{a}$ (mdyn/Å)	Cl-I⋯O^−^-N^+^	$k_{n}^{a}$ (mdyn/Å)
1	O-1	4.866	O-2	4.738
2	Q	4.916	P-2	4.785
3	P-1	4.944	M	4.867
4	L-1	5.061	L-2	4.896
5	N-1	6.829	N-2	5.297

The local mode frequencies $\omega_{n}^{a}$ for N^+^-O^−^ bond stretching can be calculated by $\omega_{n}^{a}=\sqrt{k_{n}^{a}}\cdot 476.8$, where $k_{n}^{a}$ is in the unit of mdyn/Å and the resulting vibrational frequency $\omega_{n}^{a}$ is in the unit of cm${}^{-1}$.

## Supplementary Materials

The following are available online at https://www.mdpi.com/1420-3049/25/7/1589/s1, Cartesian coordinates of all optimized primitive cell structures in this work.

## Abbreviations

The following abbreviations are used in this manuscript:

X	Halogen
ESP	Electrostatic Potential
SAPT	Symmetry-Adapted Perturbation Theory
CSD	Cambridge Structural Database
COD	Crystallography Open Database
VASP	Vienna Ab initio Simulation Package
PES	Potential Energy Surface
NBO	Natural Bond Orbital
DFT	Density Functional Theory
